# Enhanced Soft Sensor with Qualified Augmented Samples for Quality Prediction of the Polyethylene Process

**DOI:** 10.3390/polym14214769

**Published:** 2022-11-07

**Authors:** Yun Dai, Angpeng Liu, Meng Chen, Yi Liu, Yuan Yao

**Affiliations:** 1 Institute of Process Equipment and Control Engineering, Zhejiang University of Technology, Hangzhou 310023, China; 2 Guangdong Basic and Applied Basic Research Foundation, Guangzhou 510640, China; 3 Department of Chemical Engineering, National Tsing Hua University, Hsinchu 30013, Taiwan

**Keywords:** soft sensor, polymerization process, data augmentation, data selection, generative adversarial network, support vector regression

## Abstract

Data-driven soft sensors have increasingly been applied for the quality measurement of industrial polymerization processes in recent years. However, owing to the costly assay process, the limited labeled data available still pose significant obstacles to the construction of accurate models. In this study, a novel soft sensor named the selective Wasserstein generative adversarial network, with gradient penalty-based support vector regression (SWGAN-SVR), is proposed to enhance quality prediction with limited training samples. Specifically, the Wasserstein generative adversarial network with gradient penalty (WGAN-GP) is employed to capture the distribution of the available limited labeled data and to generate virtual candidates. Subsequently, an effective data-selection strategy is developed to alleviate the problem of varied-quality samples caused by the unstable training of the WGAN-GP. The selection strategy includes two parts: the centroid metric criterion and the statistical characteristic criterion. An SVR model is constructed based on the qualified augmented training data to evaluate the prediction performance. The superiority of SWGAN-SVR is demonstrated, using a numerical example and an industrial polyethylene process.

## 1. Introduction

Data-driven soft sensor models [1,2,3,4,5,6,7] have been applied extensively to provide important real-time information for the quality prediction of industrial polymerization processes in modern industry [8,9]. Although without an in-depth understanding of the process mechanisms, data-driven soft sensors have been constructed for difficult-to-measure product quality using easy-to-measure process measurements. Various soft sensors have received increasing attention in recent years, including partial least squares regression [10,11], Gaussian process regression [12,13], support vector regression (SVR) [14,15,16], and neural networks [17,18,19,20,21]. Among them, SVR-based soft sensors have exhibited good performance in several nonlinear regression tasks.

The reliability of training data for the efficient development of data-driven soft sensors is a key aspect [22,23]. However, only limited labeled samples are obtained in many polyethylene processes, which is a phenomenon that has received less attention than it deserves. For example, in the case of frequent changes in operating conditions, manual operations result in large measurement intervals and long settling times. Consequently, the acquisition of sufficient training samples is intractable [24,25,26]. With limited available training data, it is difficult to capture the process characteristics and model the relationship between product quality and operating conditions. Hence, the development of a soft sensor model with insufficient data requires further investigation.

The virtual sample generation (VSG) technique is effective in handling the problem of the insufficient construction of soft sensors with limited training data [27,28,29,30]. Several VSG methods have been developed for data augmentation, which can be divided into three types: sampling-based, information diffusion-based, and deep learning-based VSGs. For sampling-based VSG, a typical method named bootstrap [31] has been increasingly adopted for data augmentation, owing to its simple mechanism. Nevertheless, as copies of the original samples are generated, the virtual samples that are obtained via bootstrap may not carry new information and cannot fill the gaps between samples. Information diffusion-based VSG is based on the distribution function of the sample space. Typical methods include mega-trend diffusion [32] and tree-based trend diffusion [33]. These two methods employ the information diffusion principle to derive the diffusion function and generate new samples using the fuzzy set theory. However, an appropriate diffusion function and coefficient cannot easily be determined.

Deep learning-based VSG methods have gained increasing attention in fields such as imaging and natural language processing [34]. In recent years, deep learning-based VSG methods have also been adopted in the process industry [35]. The generative adversarial network (GAN) [36,37,38,39], as one promising generative model, has been well studied and is valued for its generative properties. By generating virtual data that resemble actual data, the GAN enlarges the sample capacity to enhance the prediction performance. Although various improvements have been made in GANs, including alternative loss functions and training strategies, the training process remains unstable [40,41,42,43]. Therefore, the quality of the generated samples remains uncertain. In practice, both suitable and unsuitable data exist simultaneously among the generated candidates. The prediction performance of the model will deteriorate if unsuitable virtual samples are included in the training set. Hence, data selection for the total number of virtual candidates is significant. Jiang and Ge [42] used the Mahalanobis and Euclidean distances to measure the similarity between different samples and, subsequently, selected qualified candidates for data augmentation. However, they focused on the original individual samples separately for data selection, resulting in the dilemma of local minima. Thus, a new data selection strategy that considers the general distribution is necessary.

This study aims to develop an enhanced data augmentation soft sensor framework to meet the challenge of limited labeled samples in the polyethylene process. The proposed soft sensor is named the selective Wasserstein GAN, with gradient penalty-based SVR (SWGAN-SVR). First, to expand the sample capacity and enrich the data information, virtual samples are generated using a Wasserstein GAN with a gradient penalty (WGAN-GP) network. Owing to the instability of the model training, both suitable and unsuitable samples are generated for the task simultaneously. Subsequently, a data selection strategy is adopted for sample filtering, which is composed of two parts: the centroid metric criterion and the statistical characteristic criterion. Moreover, the selected qualified samples serve as supplements for the original samples. Without the loss of generality, SVR is adopted as the base regression model. Consequently, using qualified augmented virtual samples, a more accurate and reliable prediction model can be constructed, compared to that using only the original data.

The remainder of this paper is organized as follows. Section 2 briefly introduces the preliminaries. In Section 3, the SWGAN-SVR soft sensor is presented, along with its algorithmic implementation. Section 4 demonstrates the effectiveness of SWGAN-SVR in the polyethylene process. Finally, our concluding remarks are presented in Section 5.

## 2. Preliminaries

### 2.1. Problem Statement

**Figure 1:**
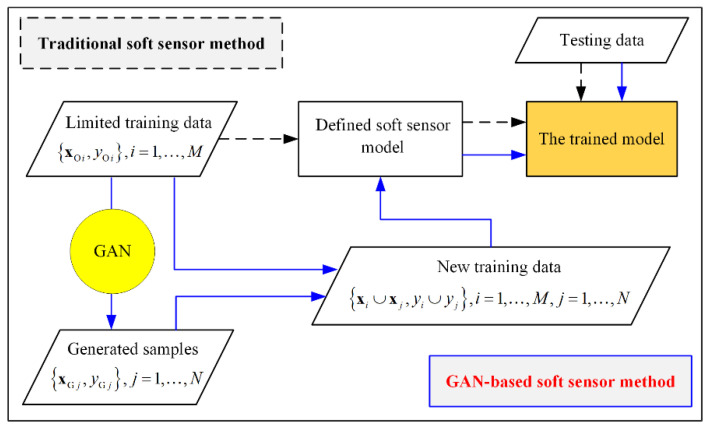
Flowchart of traditional and GAN-based soft sensor frameworks.

It has traditionally been assumed that the amount of available training data is sufficient for soft sensor modeling, and many studies have focused on the design and improvement of modeling methods. However, it is difficult to collect sufficient samples in many situations, such as for industrial processes with large measurement intervals or during the early stages of new working conditions [5]. GANs, which are unsupervised generative models, have been adopted to generate virtual samples for data augmentation. The connection and main differences between traditional supervised soft sensor models and data-augmentation-based candidates are illustrated in Figure 1. Traditional methods use only the available limited labeled samples, which are denoted as $\left\{Z_{O}\right\}=\left\{X_{O},Y_{O}\right\}$ for the purposes of model construction, where $\left\{X_{O}\right\}={\left\{x_{Oi}\right\}}_{i=1,\ldots ,M}$ and $\left\{Y_{O}\right\}={\left\{y_{Oi}\right\}}_{i=1,\ldots ,M}$ represent the input and output data with *M* samples, respectively. In contrast, data-augmentation-based soft sensors are constructed based on the augmented dataset. To address the problem of insufficient models that are established with limited training data, virtual samples, which are denoted as $\left\{Z_{G}\right\}=\left\{X_{G},Y_{G}\right\}$, are generated using a GAN, where $\left\{X_{G}\right\}={\left\{x_{Gi}\right\}}_{j=1,\ldots ,N}$ and $\left\{Y_{G}\right\}={\left\{y_{Gi}\right\}}_{j=1,\ldots ,N}$ are the input and output data, respectively, with *N* generated samples.

Unfortunately, owing to the unstable training of GANs [40,41,42,43], unsuitable samples are inevitably generated. It is worth noting that the distributions of unsuitable samples do not properly match the distribution of the real data. If these unsuitable samples are merged with the original data for establishing the model, the prediction of the soft sensor may be degraded. In this study, a data selection strategy is proposed to improve the quality of the generated samples. It is expected that a more reliable soft sensor model can be obtained by introducing these newly qualified virtual samples into the training data.

### 2.2. WGAN-GP Data Augmentation Approach

GANs have recently attracted significant attention owing to their good distribution-learning capabilities. The vanilla GAN uses the Jensen–Shannon (JS) divergence to measure the distance between the generated and original data. However, this often causes problems, such as mode collapse and vanishing gradients [44]. To address these problems, Arjovsky et al. proposed the WGAN [44], which uses the Earth-Mover distance rather than JS divergence as a distance measurement. In the WGAN, to enforce the Lipschitz constraint, the weights of the discriminator are clipped to lie within a compact space [−*r*, *r*], where *r* is a constant. The discriminator attempts to distinguish between the real and generated samples and concentrates its parameter distribution on the two extremes of the maximum and minimum; that is, *r* and −*r*. Consequently, a WGAN often becomes stuck in a poor regime and fails to learn.

To solve the problem caused by the weight clipping of the WGAN, Gulrajani et al. proposed a WGAN with a gradient penalty (WGAN-GP) [45]. Specifically, a penalty constraint is imposed on the gradient norm of the discriminator. The weight of the discriminator is reduced to an extremely small range using the gradient penalty strategy, which accelerates the model convergence and solves the gradient explosion problem. The objective function of the WGAN-GP is as follows:

(1) $$\begin{array}{cc} \underset{G}{\min}\underset{D}{\max}V\left(D,G\right)= & \underset{\mathrm{original}\;\mathrm{WGAN}}{\underbrace{E_{z_{0}∼p_{\mathrm{data}}\left(z_{O}\right)}\left[D\left(z_{O}\right)\right]-E_{z_{G}∼p_{G}\left(z_{G}\right)}\left[D\left(z_{G}\right)\right]}}\\ & \underset{\mathrm{the}\;\mathrm{penalty}\;\mathrm{term}}{\underbrace{-\lambda E_{\hat{z}∼p_{\hat{z}}}\left[{\left(∥\nabla_{\hat{z}}D\left(\hat{z}\right){∥}_{2}-1\right)}^{2}\right]}} \end{array},$$

where λ is the penalty coefficient, $\hat{z}$ is sampled through random interpolation on the connecting line of the original data $z_{O}$ and generated data $z_{G}$; that is, $\hat{z}=\theta z_{O}+(1-\theta )z_{G}$, and θ is a random number in [0, 1].

## 3. The SWGAN-Based Soft Sensor Framework

### 3.1. Virtual Sample Selection Strategy

**Figure 2:**
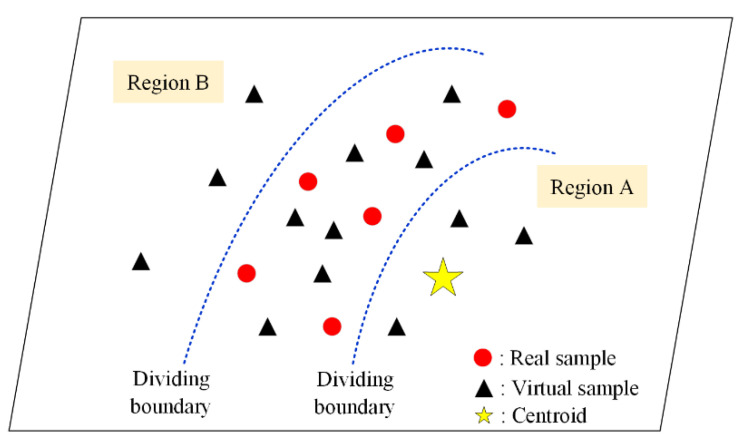
Distribution scattering of the real and virtual samples.

Owing to the unstable training of the WGAN-GP, the quality of the generated virtual samples varies significantly. A data selection strategy is proposed for sample filtering, to eliminate the negative effects of unqualified virtual samples that are generated by the WGAN-GP for model construction. The selection strategy includes a centroid metric criterion, which is denoted as S1, and a statistical characteristic criterion, which is denoted as S2. The distribution scatters of the original and rough virtual samples are plotted in Figure 2. The distribution of most virtual samples conforms to the real data distribution. However, the WGAN-GP also generates samples that are located in regions A and B, which are far from the distribution of the original data. If the generated samples in regions A and B, which are regarded as unqualified, are added to the original set, the prediction performance of the model may deteriorate. A detailed description of the proposed selection strategy is provided below.

First, the S1 criterion is developed to filter the virtual samples in region A. The samples in region A are too close to the centroid $z_{C}$ of the original samples. Furthermore, the distribution of these samples is not uniform compared to that of the original sample. Thus, the virtual samples around the centroid are considered to be information-poor and unqualified. The centroid $z_{C}$ is defined as the closest point in space to the original data, as follows:

(2) $$z_{C}=(\mu_{\mathrm{OX}},\mu_{OY})=\frac{1}{M}(\sum_{i=1}^{M}x_{Oi},\sum_{i=1}^{M}y_{Oi}),$$

where $\mu_{\mathrm{OX}}$ and $\mu_{OY}$ are the process and target variables of $z_{C}$, respectively.

The Euclidean distance is commonly used to measure the distance between two samples. A large distance indicates that the samples are far from one another. The square of the distance between $z_{C}$ and a finite number of original samples is formulated as follows:

(3) $$d_{C}=\sum_{i=1}^{M}{\left\|z_{C}-z_{Oi}\right\|}_{2}^{2}=\min(\sum_{i=1}^{M}{\left\|z_{r}-z_{Oi}\right\|}_{2}^{2}),$$

where $z_{Oi}$ is the *i*^th^ original sample and $z_{Oi}=(x_{Oi},y_{Oi})$, and $z_{r}$ is any point in space.

Similarly, the square of the distance between the *j*^th^ generated sample and the original samples is calculated as follows:

(4) $$d_{j}=\sum_{i=1}^{M}{\left\|z_{Gj}-z_{Oi}\right\|}_{2}^{2},$$

where $z_{Gj}=(x_{Gj},y_{Gj})$ is the *j*^th^ generated sample.

According to the definitions of $d_{j}$ and $d_{C}$, $d_{j}\geq d_{C}$. A smaller $d_{j}$ means that $z_{Gj}$ is closer to $z_{C}$, indicating a more dissimilar distribution of $z_{Gj}$ to the original samples. A sample in region A satisfies $d_{j}<\rho d_{C}$, where ρ≥1 is a parameter. Therefore, the qualified samples, based on the S1 criterion, are defined as:

(5) $$d_{j}\geq \rho d_{C},\rho \geq 1.$$

Subsequently, the S2 criterion is adopted to filter the unsuitable virtual samples in area B. The samples in region B are far away from the distribution of the original data and tend to be outliers. The samples can be screened according to the statistical characteristics of the original samples. Based on the probability density function p(x) for each normal operating data point of the initial samples, the 100β% confidence bound can be defined as the likelihood threshold *h* that satisfies the following formula:

(6) $$\int_{x:p(x)>h}p(x)dx=\beta ,$$

where p(x) is a multivariate Gaussian distribution and the above confidence bounds can be found in a previous paper [46]. In particular, when the generated sample $x_{Gj}$ satisfies the following formula, it is considered as an outlier, as follows:

(7) $$D^{2}={\left(x_{Gj}-\mu_{\mathrm{OX}}\right)}^{T}C_{\mathrm{OX}}^{-1}(x_{Gj}-\mu_{\mathrm{OX}})>\chi_{q}^{2}(\beta ),$$

where $C_{\mathrm{OX}}^{-1}$ is the covariance of the input data of the original samples and $\chi_{q}^{2}(\beta )$ is the *β*-fractile of the Chi-square distribution, with a degree of freedom, *q*.

In summary, according to the aforementioned two-stage data selection strategy, *k*-qualified samples are selected from the rough generated data and are denoted as ${\left\{x_{Sj},y_{Sj}\right\}}_{j=1,\ldots ,k}$. This data selection strategy makes the selected virtual samples more homogeneous, in agreement with the original data distribution.

### 3.2. SWGAN-SVR Soft Sensor Model

In this case, SVR is adopted as the base soft-sensor model for nonlinear processes. SVR is a statistical learning method that uses the structural risk-minimization criterion instead of the empirical risk-minimization criterion for model construction [15]. The target function of the SVR is as follows [15]:

(8) $$\begin{array}{l} \min J(w,b,\xi_{i},\xi_{i}^{*})=\frac{1}{2}{\left\|w\right\|}^{2}+\gamma \sum_{i=1}^{n}\left(\xi_{i}+\xi_{i}^{*}\right)\\ s.t.\;\left\{\begin{array}{c} y_{i}-w^{T}\phi (x_{i})-b\leq \varepsilon +\xi_{i}\\ w^{T}\phi (x_{i})+b-y_{i}\leq \varepsilon +\xi_{i}^{*}\\ \xi_{i},\xi_{i}^{*}\geq 0,i=1,\ldots ,n \end{array}\right. \end{array},$$

where b is the bias, w is the weight vector, $\xi_{i}$ and $\xi_{i}^{*}$ are slack variables, γ is a regularization parameter that controls the penalty for samples exceeding the fitting error, ϕ is a nonlinear kernel function, ε is an insensitivity coefficient, and *n* is the number of samples for the SVR model.

The constrained optimization can be solved using the Lagrange function by introducing Lagrange multipliers. Subsequently, Equation (8) is converted into a dual problem, as follows:

(9) $$\begin{array}{l} \underset{\alpha ,\alpha^{*}}{\min}\frac{1}{2}\sum_{i=1}^{n}\sum_{j=1}^{n}\left(\alpha_{i}^{*}-\alpha_{i})(\alpha_{j}^{*}-\alpha_{j})K(x_{i},x_{j}\right)+\varepsilon \sum_{i=1}^{n}\left(\alpha_{i}^{*}+\alpha_{i}\right)-\sum_{i=1}^{n}\left(\alpha_{i}^{*}-\alpha_{i}\right)\\ s.t.\left\{\begin{array}{c} \sum_{i=1}^{n}\left(\alpha_{i}^{*}-\alpha_{i}\right)=0\\ 0\leq \alpha_{i},\alpha_{i}^{*}\leq \gamma ,i=1,2,\ldots ,n \end{array}\right. \end{array},$$

where $\alpha_{i}$ and $\alpha_{i}^{*}$ are the Lagrange multipliers and K(⋅, ⋅) represents a kernel function. In this study, the radial basis function (RBF) is adopted:

(10) $$K(x_{i},x_{j})=\exp(-\psi {\left\|x_{i}-x_{j}\right\|}^{2}),$$

where ψ>0 is a controlling parameter for the RBF kernel width.

Therefore, the SVR model can be described as

(11) $$f(x)=\sum_{i=1}^{n}(\alpha_{i}^{*}-\alpha_{i})K(x_{i},x)+b,$$

**Figure 3:**
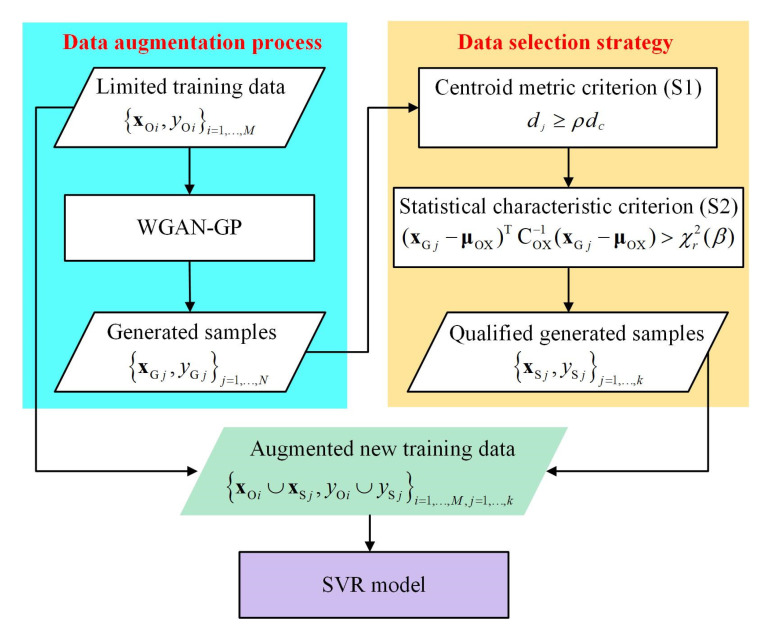
Flowchart of the proposed SWGAN-SVR method.

A flowchart of the SWGAN-SVR model is presented in Figure 3. It is difficult to develop a reliable SVR soft sensor for the initial limited training data, ${\left\{x_{Oi},y_{Oi}\right\}}_{i=1,\ldots ,M}$. In such a situation, the WGAN-GP is adopted for data augmentation and *N* virtual samples are generated, which is denoted as ${\left\{x_{Gj},y_{Gj}\right\}}_{j=1,\ldots ,N}$. Furthermore, considering the unstable training process of the WGAN-GP, unsuitable virtual samples are generated, which need to be screened out from the group of rough virtual samples. Consequently, after employing the proposed two-stage data selection strategy; that is, the centroid metric criterion S1 and statistical characteristic criterion S2, *k*-qualified samples ${\left\{x_{Sj},y_{Sj}\right\}}_{j=1,\ldots ,k}$ are obtained. By combining qualified virtual samples with the initial limited training samples, a new augmented training sample set is obtained, which can be denoted as ${\left\{x_{O}{}_{i}\cup x_{S}{}_{j},y_{O}{}_{i}\cup y_{Sj}\right\}}_{i=1,\ldots ,M,j=1,\ldots ,k}$. Subsequently, an SVR soft sensor is constructed for quality prediction. Note that other supervised soft-sensor modeling methods, such as partial least squares regression and Gaussian process regression, can also replace SVR in this framework.

## 4. Results and Discussion

A numerical example and an industrial polyethylene process were adopted to validate the effectiveness of the proposed SWGAN-SVR modeling method. The commonly used root-mean-square error (RMSE), coefficient of determination ($R^{2}$), and mean absolute error (MAE) indices were used for the performance evaluation and are expressed as follows:

(12) $$\mathrm{RMSE}=\sqrt{\frac{1}{m}\sum_{t=1}^{m}(y_{t}-{\hat{y}}_{t}{)}^{2}},$$

(13) $$R^{2}=1-\sum_{t=1}^{m}{\left(y_{t}-{\hat{y}}_{t}\right)}^{2}/\sum_{t=1}^{m}{\left(y_{t}-{\bar{y}}_{t}\right)}^{2},$$

(14) $$\mathrm{MAE}=\frac{1}{m}\sum_{t=1}^{m}\left|y_{t}-{\hat{y}}_{t}\right|,$$

where $y_{t}$ and ${\hat{y}}_{t}$ are the quality measurement and prediction values of the *t*^th^ observation, respectively, and *m* is the sample size.

### 4.1. Numerical Example

A numerical example with a two-dimensional input and one-dimensional output was constructed to simulate the process of insufficient initial training samples:

(15) $$\begin{array}{l} x_{1}=3u^{2}+4u,\;u=-10,-9.8,-9.6,\ldots ,10\\ x_{2}=8u+2\cos(\pi u/3),\;u=-10,-9.8,-9.6,\ldots ,10\\ y=x_{1}+x_{2}+e \end{array},$$

where $x_{1}$ and $x_{2}$ are two state variables that are constructed using the variable u, y is an output variable, and e is Gaussian noise with a zero mean and a variance of 0.01.

In this study, 100 samples were collected. To build the soft-sensor model, 50 samples were randomly selected as the training data and 50 samples were used for testing. In such a situation, using only limited training samples to train an SVR soft sensor may be insufficient. Therefore, it is essential to generate virtual samples to increase the data capacity and enrich the data diversity.

**Figure 4:**
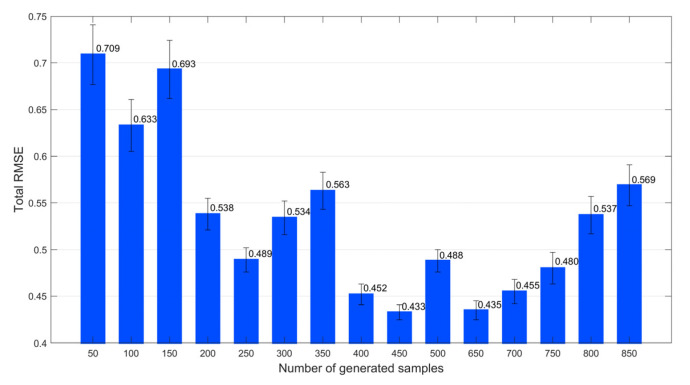
Total RMSE values of the different numbers of generated samples for the numerical example.

First, we investigated the number of generated samples that were sufficient for this example, using a 10-fold cross-validation algorithm. Specifically, a new training set containing both the original samples and generated virtual samples was divided into 10 non-overlapping subsets. Subsequently, based on the *i*^th^ subset, which was regarded as a temporary test set, and extra subsets other than the *i*^th^ subset, which was regarded as a temporary training set, an SVR model was constructed. Each subset was used as a temporary test set, in turn. Consequently, the total prediction result for a certain number of generated samples was obtained across 10 trials. The RMSE results for different numbers of generated samples are depicted in Figure 4. As the number of virtual samples increased, the RMSE value first decreased and then increased. This is mainly because the generated virtual samples filled the information gap in the initial training stage, which improved the model prediction accuracy. When the size of the virtual samples was sufficiently large, the influence of the initial samples was weakened, and more significant differences occurred between the initial and virtual samples. Therefore, as illustrated in Figure 4, the appropriate number of virtual samples for this example was 450.

**Figure 5:**
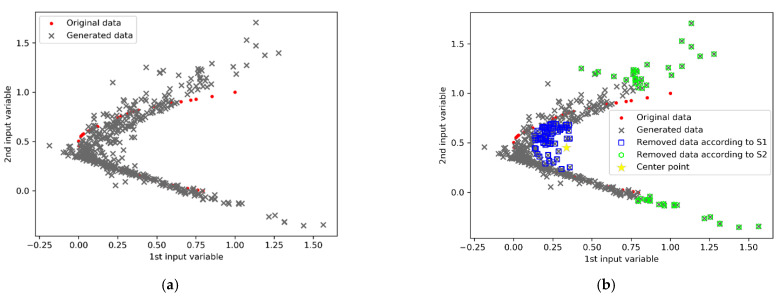
Scatter distributions for the numerical example: (**a**) original data and rough generated data, and (**b**) qualified virtual samples.

The scatter distributions of the original samples and the 450 generated samples are presented in Figure 5a. Several unsuitable samples did not conform to the initial data distribution. According to the proposed S1 and S2 data selection criteria, the scatter distribution of the qualified virtual samples, rough virtual samples, and initial limited samples are shown in Figure 5b. Unsuitable samples that were too close to the centroid and distant outliers were filtered. Consequently, the qualified virtual samples matched the distribution of the original samples. When combined with the original training data, the qualified virtual samples served as complements to the initial samples. The SWGAN-SVR model was built, based on the qualified augmented training samples; the prediction results for the test set are listed in Table 1. For comparison, the prediction results of SVR, WGAN-SVR, and WGAN-SVR using the S1 criterion (denoted as WGAN-SVR(S1)), and WGAN-SVR using the S2 criterion (denoted as WGAN-SVR(S2)), are also listed in Table 1. WGAN-SVR, WGAN-SVR(S1), WGAN-SVR(S2), and SWGAN-SVR outperformed the SVR method, with smaller RMSE and MAE values and larger R^2^ values. This is mainly because the generated samples increased the diversity of the training samples. The prediction performances of WGAN-SVR(S1) and WGAN-SVR(S2) were further enhanced, compared to the results of WGAN-SVR. By adopting only one data selection criterion, unsuitable virtual samples around the centroid or far-away outliers were screened out, which improved the quality of the augmented samples. This also demonstrates that unsuitable virtual samples result in the insufficient construction of reliable soft sensors. Furthermore, after simultaneously adopting the S1 and S2 criteria, SWGAN-SVR achieved the best prediction performance among the five methods. This indicates that a two-stage data selection strategy is beneficial for selecting qualified augmented samples and improving the performance of the base SVR soft sensor.

**Figure 6:**
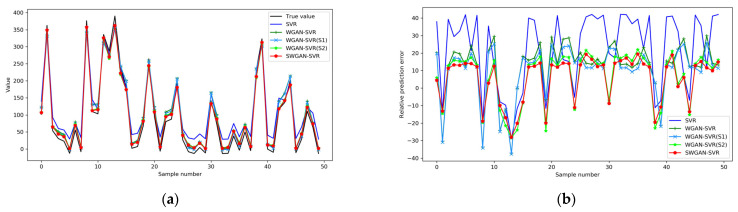
Prediction performance of five methods for the numerical example: (**a**) the assay and predicted values, and (**b**) the relative prediction errors.

**Figure 7:**
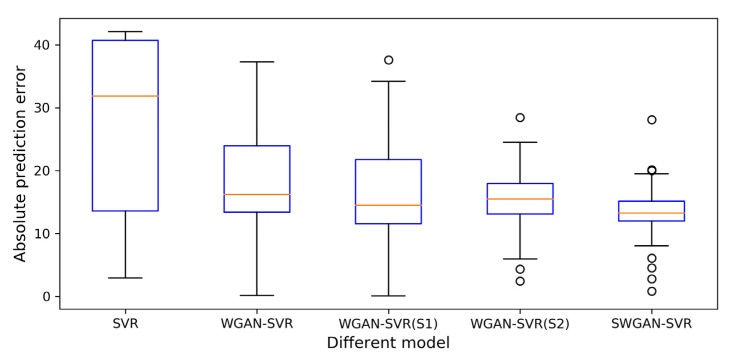
Absolute values of the prediction errors for the numerical example.

For a better illustration, the detailed prediction results and relative prediction errors of the five soft sensors on the test set are presented in Figure 6a,b, respectively. As shown in Figure 6a, SWGAN-SVR tracked the real trajectory better than the other four soft sensors, and the prediction curve of SWGAN-SVR was the one that was most consistent with the real curve. As illustrated in Figure 6b, the prediction errors of the proposed SWGAN-SVR were much smaller for the entire test set, and the errors were mostly around zero. A boxplot of the absolute prediction error values for the five methods is shown in Figure 7. SWGAN-SVR had a narrower error range, which was closer to zero, than the other four methods. Furthermore, as demonstrated through a comparison of the red lines in the boxes, the median value of the absolute error was smaller than that of the other four methods, indicating a better prediction performance for SWGAN-SVR.

### 4.2. Industrial Polyethylene Process

An industrial polyethylene process [4] was utilized to verify the necessity and superiority of the proposed method for practical applications. The product of the polyethylene manufacturing process was sampled once daily from the laboratory. Hence, in the initial stage of a new product grade, the collected quality variables (that is, the melt index (MI)) are insufficient for the development of a reliable soft sensor. After using a simple 3-sigma criterion to remove outliers, 60 samples were investigated. The dataset was partitioned into two parts: 30 randomly selected samples were used as the training data and the remaining 30 samples were used for testing.

**Figure 8:**
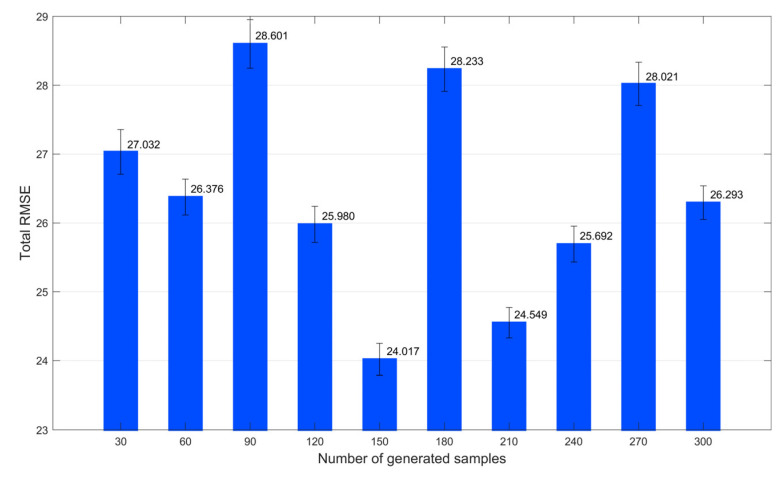
Total RMSE values of the different numbers of generated samples for the polyethylene process.

Using a 10-fold cross-validation method, a suitable number of virtual samples was first determined for this example. The complete RMSE indices for different numbers of virtual samples are presented in Figure 8. The RMSE value was smallest when the number of generated samples was 150. Hence, 150 virtual samples were generated as an appropriate supplement to the initial limited samples. The proposed data selection strategy was adopted to improve the quality of the generated virtual samples. Subsequently, the proposed SWGAN-SVR model was built, based on the qualified augmented samples. Furthermore, SVR, WGAN-SVR, WGAN-SVR(S1), and WGAN-SVR(S2) were built to predict the MI value. The details of the prediction performance of the five methods on the test set are listed in Table 2. According to the prediction results, the SVR method achieved the largest RMSE value and smallest R^2^ value, indicating the worst prediction accuracy among the five methods. This occurred because the initial training data were insufficient for the construction of reliable soft sensors. With this data augmentation strategy, the WGAN-SVR, WGAN-SVR(S1), WGAN-SVR(S2), and SWGAN-SVR methods can improve the prediction accuracy, compared to the SVR approach. The generated virtual samples fill the information gap in the initial data and increase the sample capacity. Moreover, by adopting the two-stage data selection criteria, the SWGAN-SVR method achieved the best prediction performance among the five methods. The SWGAN-SVR method attempts to select the qualified virtual samples and, subsequently, to improve the quantity and quality of the initial training data. Note that owing to the strong nonlinearity of this example, the R^2^ index was relatively smaller than that of the numerical example described in Section 4.1.

**Figure 9:**
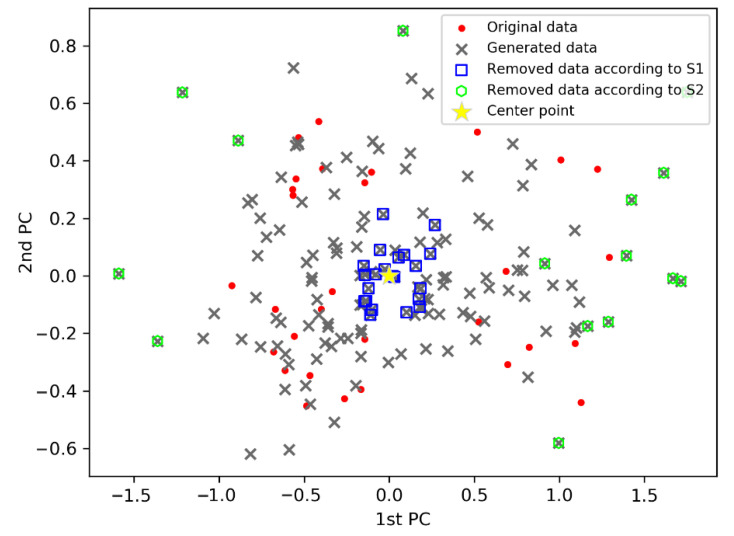
Scatter distribution comparison of the qualified virtual samples for the polyethylene process.

The scatter distribution of the rough generated samples and selected unsuitable samples are presented in Figure 9. Virtual samples close to the centroid and the distant outliers were filtered. The remaining qualified samples matched well with the distribution of the original samples. Moreover, the diversity of the original samples increased with the incorporation of the qualified samples.

**Figure 10:**
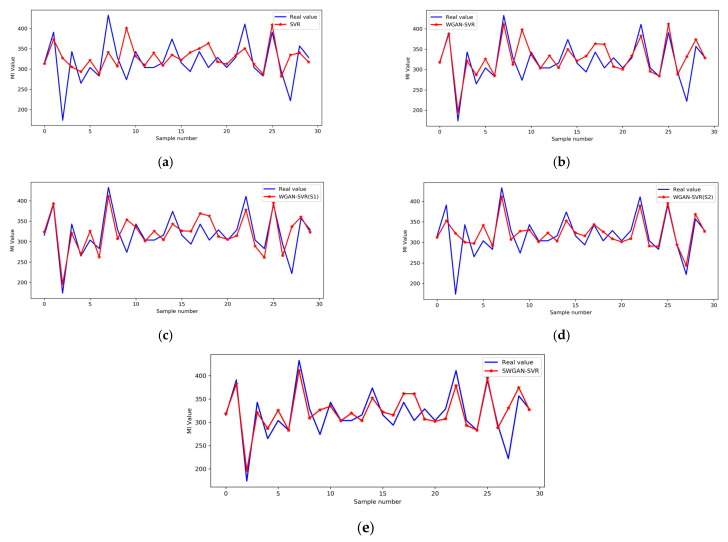
Assay and predicted values for the polyethylene process: (**a**) SVR, (**b**) WGAN-SVR, (**c**) WGAN-SVR(S1), (**d**) WGAN-SVR(S2), (**e**) SWGAN-SVR.

**Figure 11:**
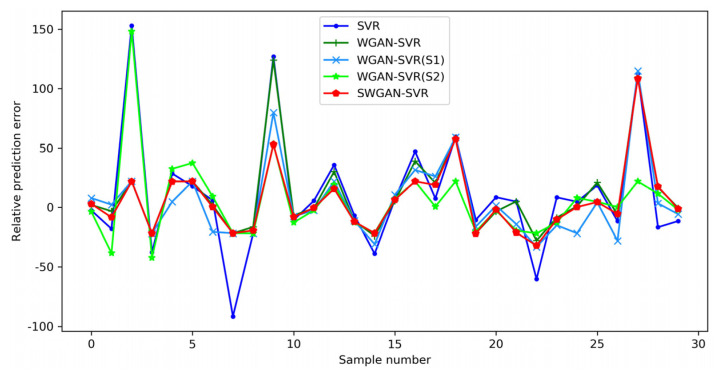
Relative prediction errors for the polyethylene process.

The detailed prediction results of the five soft sensors on the test set are depicted in Figure 10. The proposed SWGAN-SVR method was superior to the other four methods in terms of tracking the real trend of the output variable. The prediction of SWAGN-SVR was in good agreement with the actual trajectory of the MI value, and, thus, exhibited a much smaller deviation. The relative prediction errors of the five methods are shown in Figure 11. The SWGAN-SVR method achieved the best prediction performance and yielded the smallest prediction error at most sampling points. Consequently, the obtained results indicate that the proposed SWGAN-SVR soft sensor can enhance prediction performance when dealing with insufficient training samples.

## 5. Conclusions

In this study, a reliable soft sensor framework is developed to enhance prediction performance by introducing augmented data. Because having limited training data will be insufficient for establishing a reliable soft sensor, rough virtual samples are generated using the WGAN-GP method to enrich the sample information. Subsequently, based on a two-stage data selection strategy, qualified augmented samples are gradually selected to eliminate the negative effects of unsuitable samples on the prediction performance. Based on the qualified augmented training samples, the SWGAN-SVR method is designed to capture the process characteristics, which is beneficial for regression. The prediction results for the two examples demonstrate the advantages of the proposed approach. Further investigations will aim to enhance the quality of the generated samples, using GANs. Additionally, the combination of the process characteristics to generate more informative samples for practical applications is an interesting topic.

## Figures and Tables

**Table 1:** Performance comparison of SWGAN-SVR and other methods for the numerical example.

	RMSE	R^2^	MAE
SVR	30.399	0.933	27.248
WGAN-SVR	20.083	0.971	18.548
WGAN-SVR(S1)	18.222	0.976	16.653
WGAN-SVR(S2)	16.249	0.981	15.478
SWGAN-SVR	**14.144**	**0.986**	**13.407**

**Table 2:** Performance comparison of SWGAN-SVR and other methods for industrial polyethylene.

	RMSE	R^2^	MAE
SVR	50.925	0.015	33.061
WGAN-SVR	36.227	0.502	22.550
WGAN-SVR(S1)	32.923	0.588	22.835
WGAN-SVR(S2)	34.597	0.546	21.828
SWGAN-SVR	**28.854**	**0.684**	**19.379**

## Data Availability

The data presented in this study are available on request from the corresponding author.
